# Menopausal hormone therapy and central nervous system tumors: Danish nested case-control study

**DOI:** 10.1371/journal.pmed.1004321

**Published:** 2023-12-19

**Authors:** Nelsan Pourhadi, Amani Meaidi, Søren Friis, Christian Torp-Pedersen, Lina S. Mørch

**Affiliations:** 1 Danish Dementia Research Centre, Department of Neurology, Copenhagen University Hospital—Rigshospitalet, Copenhagen, Denmark; 2 Cancer Surveillance and Pharmacoepidemiology, Danish Cancer Institute, Danish Cancer Society, Copenhagen, Denmark; 3 Department of Public Health, University of Copenhagen, Copenhagen, Denmark; 4 Department of Cardiology, Nordsjællands Hospital, Hillerød, Denmark; Vanderbilt University School of Medicine, UNITED STATES

## Abstract

**Background:**

Use of estrogen-containing menopausal hormone therapy has been shown to influence the risk of central nervous system (CNS) tumors. However, it is unknown how the progestin-component affects the risk and whether continuous versus cyclic treatment regimens influence the risk differently.

**Methods and findings:**

Nested case-control studies within a nationwide cohort of Danish women followed for 19 years from 2000 to 2018. The cohort comprised 789,901 women aged 50 to 60 years during follow-up, without prior CNS tumor diagnosis, cancer, or contraindication for treatment with menopausal hormone therapy. Information on cumulative exposure to female hormonal drugs was based on filled prescriptions. Statistical analysis included educational level, use of antihistamines, and use of anti-asthma drugs as covariates. During follow-up, 1,595 women were diagnosed with meningioma and 1,167 with glioma. The median (first–third quartile) follow-up time of individuals in the full cohort was 10.8 years (5.0 years to 17.5 years). Compared to never-use, exposure to estrogen-progestin or progestin-only were both associated with increased risk of meningioma, hazard ratio (HR) 1.21; (95% confidence interval (CI) [1.06, 1.37] *p* = 0.005) and HR 1.28; (95% CI [1.05, 1.54] *p* = 0.012), respectively. Corresponding HRs for glioma were HR 1.00; (95% CI [0.86, 1.16] *p* = 0.982) and HR 1.20; (95% CI [0.95, 1.51] *p* = 0.117). Continuous estrogen-progestin exhibited higher HR of meningioma 1.34; (95% CI [1.08, 1.66] *p* = 0.008) than cyclic treatment 1.13; (95% CI [0.94, 1.34] *p* = 0.185). Previous use of estrogen-progestin 5 to 10 years prior to diagnosis yielded the strongest association with meningioma, HR 1.26; (95% CI [1.01, 1.57] *p* = 0.044), whereas current/recent use of progestin-only yielded the highest HRs for both meningioma 1.64; (95% CI [0.90, 2.98] *p* = 0.104) and glioma 1.83; (95% CI [0.98, 3.41] *p* = 0.057). Being an observational study, residual confounding could occur.

**Conclusions:**

Use of continuous, but not cyclic estrogen-progestin was associated with increased meningioma risk. There was no evidence of increased glioma risk with estrogen-progestin use. Use of progestin-only was associated with increased risk of meningioma and potentially glioma. Further studies are warranted to evaluate our findings and investigate the influence of long-term progestin-only regimens on CNS tumor risk.

## Abstract 2

In this Danish nested case-control study, Nelsan Pourhadi and colleagues investigate the influence of the progestin-component of menopausal hormone therapy on central nervous system tumors.

## Author summary

**Why was this study done?:**

- Meningioma is the most common brain tumor, typically benign, while glioma is the most common malignant brain tumor. Meningioma is more common in women compared to men, whereas it is the opposite for glioma, suggestive of sex-related risk factors such as female sex hormones (estrogen and progestin).
- Previous research found use of hormone therapy for menopausal symptoms to be linked to increased meningioma risk, but there are inconsistent findings regarding glioma risk.
- The influence of progestin (for perimenopausal bleeding disturbances) and different hormone therapies (continuous or cyclic) on brain tumor risk is currently unresolved.

**What did the researchers do and find?:**

- In a Danish nationwide population-based study, we included 789,901 women of whom 1,595 developed meningioma and 1,167 developed glioma during the study period from 2000 to 2018. Use of hormone therapies among the women was evaluated through filled prescriptions.
- Use of continuous, but not cyclic, estrogen-progestin therapy was associated with increased meningioma risk, but not of glioma.
- Use of progestin-only was associated with increased meningioma risk and possibly also glioma.

**What do these findings mean?:**

- Findings from this study contribute with knowledge on how brain tumors may be affected by female sex hormones, particularly progestin. Future studies should further investigate the role of long-term progestin use on brain tumor risk.
- Findings suggest cyclic menopausal hormone therapy as a safer treatment compared to continuous therapy regarding meningioma risk.
- Due to little usage of transdermal hormone therapy among the included women, we were not able to investigate this administration form. This is an observational study; thus, unmeasured influences cannot be ruled out.

## Introduction

Meningiomas are the most common benign tumors of the central nervous system (CNS) and occur more frequently in women, with a female:male ratio of up to 3.5, the greatest ratio being among middle-aged individuals [1]. Conversely, gliomas are the most common malignant CNS tumors, with a 50% higher incidence among men [2]. While the etiology of these tumors is largely unknown and only a few rare risk factors have been established, the sex difference in incidence indicates potential risk factors related to the sex, including exogenous use of female sex hormones [3].

Meningiomas and gliomas are hormone sensitive, and both tumors express estrogen and progestin receptors [4,5]. In observational studies, oral estrogen-only hormone therapy, which is solely recommended for hysterectomized women with vasomotor symptoms, has been associated with an increased risk of meningioma [6,7]. However, the findings with use of combined estrogen-progestin, the primary hormone therapy in menopausal women with an intact uterus, are conflicting with some studies reporting increased risk [8,9] and others no association [6]. Menopausal hormone therapy and oral contraceptives have been associated with a reduced risk of glioma in a meta-analysis [10], thus, estrogen receptors have been suggested as a potential target in endocrine treatment of glioma [11]. However, evidence is conflicting with other studies reporting of increased risk of glioma among users of systemic estrogen-only [6,12,13].

The specific influence of progestin on CNS tumor development is unresolved. While the hormone therapy-associated risk of meningioma is thought to be primarily driven by the estrogen component, emerging evidence suggests progestin as a potential independent risk factor [14,15]. A recent observational study reported a strong dose-response relationship between female use of a progestin with antiandrogen effect and risk of meningioma [15]. Another recent study found increased meningioma risk with prolonged exposure to other progestins, thereby emphasizing that the influence of progestin in meningioma development appears not to be restricted to progestins with antiandrogen effects [16]. The role of progestin in meningioma development is further indicative of a potential difference in the influence of cyclic versus continuous treatment with progestin in combined hormone therapy. However, most previous studies were not able to assess cyclic versus continuous treatments or found a similar influence of these treatments on meningioma development. Still, the studies had limited statistical precision to assess potential differential influence according to cumulative use regimens [6,8,17]. Finally, the effect of progestin on glioma risk is currently unknown.

In this nationwide population-based study, we examined the influence of menopausal hormone therapy use on incidence of meningioma and glioma focusing on estrogen-progestin use among non-hysterectomized women, with regard to type of regimen (e.g., cyclic versus continuous regimens), treatment duration, and user status. Additionally, we assessed associated incidence of meningioma or glioma with the use of estrogen-only therapy in a population of hysterectomized women.

## Methods

### Study population

By linkage of national Danish registers, we identified an open nationwide cohort of Danish female residents aged 50 to 60 years in year 2000 or turning 50 years between 2000 and 2018. Women were followed from either 1 January 2000 or from their 50th birthday during the study period from 1 January 2000 to 31 December 2018. All residents in Denmark are assigned a unique identification number registered in the Civil Registration System allowing for unambiguous linkage of data on individual level. The nationwide cohort was based on the following registers: (1) The Danish Cancer Registry [18]; (2) The National Patient Registry [19]; (3) The National Prescription Registry [20]; and (4) The Danish Education Registry [21]. A previous diagnosis of meningioma, glioma, or cancer (except non-melanoma skin cancer) led to exclusion. We also excluded women with contraindications for menopausal hormone therapy, including previous diagnosis of stroke, acute myocardial infarction, venous thrombosis, liver disease, or thrombophilia. Our main exposure of interest was combined estrogen-progestin therapy, which is used by women with an intact uterus, where the progestin component is given to protect from estrogen-induced malignant proliferation of the endometrium. Thus, hysterectomized women were excluded from the main study population, since women without a uterus should not receive combined estrogen-progestin therapy for vasomotor symptoms, but rather estrogen-only therapy. A separate cohort of hysterectomized women (with otherwise same characteristics as the non-hysterectomized main cohort) was identified to study exposure to estrogen-only therapy in its clinical target population [22]. Exclusion information (i.e., diagnoses and surgical procedures) was available from 1977. Finally, we excluded women who immigrated to Denmark after 1 January 1995 (initiation of prescription register). Women were censored during follow-up if an exclusion criterion occurred or at time of emigration or death.

Two separate nested case-control populations for meningioma and glioma were established from the nationwide cohort. Incident meningioma or glioma occurring during follow-up were considered cases. Each case was matched per birth year by incidence density matching [23,24] to 10 control individuals from the cohort who did not have a CNS tumor diagnosis or any censoring criterion at the date of case diagnosis/matching (index date). Thus, the matched populations comprised arrays of risk-sets (1,595 for meningioma and 1,167 for glioma), each consisting of 1 case and 10 controls of the same age at index date, hence, subject to the same circumstances of potential exposure to menopausal hormone therapy.

### Menopausal hormone therapy

Data on hormone therapy use in the study population was obtained from the national prescription register providing information on all redeemed prescriptions from Danish pharmacies since 1995. Hormone therapy use from 1995 and throughout follow-up was obtained from prescription records with relevant Anatomical Therapeutic Chemical (ATC) codes (Table A in S1 Text) and information on related active ingredients (estrogen or progestin type), drug unit, package size, route of administration, and date of dispensing.

Systemically administered estrogen is the primary treatment for menopausal vasomotor symptoms, and for women with intact uterus, the treatment additionally consists of a progestin for protection of the endometrium. Thus, treatment with combined estrogen-progestin was the main exposure of interest. Ever-use was defined as redeeming one or more prescriptions of a combined estrogen-progestin preparation (ATC G03F and a single preparation with ATC G03HB01) or simultaneous use of a systemic estrogen-only product (ATC G03C) and a progestin-only product (ATC G03D or the intrauterine device ATC G02BA03). Further, continuous (ATC G03FA, daily dose of progestin) and cyclic (ATC G03FB, progestin in the end of a treatment cycle) estrogen-progestin treatment regimens were identified.

To account for use of hormone preparations other than combined estrogen-progestin, we also retrieved information on (1) estrogen-only therapy; (2) progestin-only therapy (used for perimenopausal bleeding disturbances); and (3) vaginal estrogen (used to treat the genitourinary syndrome of menopause—ATC G03CA03).

Treatment duration of hormone therapy was calculated using the program “medicinMacro” in the R-package “Github/tagteam/heaven” [25,26]. Prescription information including the date of dispensing and amount of hormone therapy (package size, unit size, and number of packages) together with data on dosage recommendations from the summary of product characteristics formed the basis for the calculation. Treatment periods were calculated assuming use of the recommended default dose at initiation of treatment. If a woman redeemed additional prescriptions, the program calculated (based on up to 5 most recent prescriptions) whether treatment could be continuous assuming minimum, maximum, or default daily dose.

Women using both combined estrogen-progestin as well as another hormonal therapy product during follow-up were categorized as estrogen-progestin users. Women using both cyclic and continuous estrogen-progestin therapies were categorized as mixed users. Users of both estrogen-only and progestin-only, but not in overlapping treatment periods, were categorized separately. Women purchasing vaginal estrogen but not any systemic hormone therapy were considered a vaginal estrogen-only user. The association between vaginal estradiol use and risk of CNS tumors has been reported in a separate study [27].

### Meningioma and glioma

Meningioma was defined as the first date of validated diagnosis with meningioma obtained from the Danish Cancer Registry (International Classification of Diseases, 10th Revision (ICD-10): C70.0, C70.9, D32.0, D32.1, D32.9, D42.0, D42.1, D42.9; and International Classification of Diseases for Oncology, 3rd Edition (ICD-O-3) morphology: 95300, 95301, 95303, 95310, 95311, 95313, 95320, 95321, 95323, 95330, 95331, 95333, 95340, 95370, 95373, 95381, 95383, 95391, and 95393) [18].

Glioma was defined as the first date of validated diagnosis with glioma (including glioblastoma multiforme, astrocytoma grade II and III, and oligodendroglioma grade II and III; ICD-10: C71.0-C71.9, D33.0-D33.2, D43.0-D43.2; and ICD-O-3 morphology: 94403, 94003, 94013, 94103, 94113, 94503, 94513, 94603, 93801, 93803, 93813, 93823, 93831, 93900–94001, 94121–94401, 94413–94501).

Although glioma did not serve the purpose of an a priori-defined negative control outcome in our study, we expected different associations with the exposure (i.e., menopausal hormone therapy) compared to those for meningioma as reported in previous studies. Since the results for both CNS tumor outcomes were expected to have comparable sources of bias, any differences in associations could likely be attributed to the exposure.

### Potential confounders

Age and educational level (elementary school only, secondary school only, vocational education, university education, university education and PhD) were considered demographic and socioeconomic confounders. Due to the inverse association between allergies and CNS tumors [28,29], antihistamines (ATC R06A) and anti-asthma drugs (ATC R03) were considered potential confounders. As descriptive characteristics of the study population, we additionally included information on diabetes (antidiabetic medication), statin use, aspirin use, and NSAID use, all defined from filled prescriptions.

### Statistical analysis

Conditional logistic regression was used to calculate adjusted hazard ratios (HRs) and 95% confidence intervals (CIs) for associations between hormone therapy and meningioma or glioma in the 2 matched populations.

In the main analysis, exposure was grouped according to the type of hormone therapy: (1) No use (reference); (2) estrogen-progestin (subdivided in continuous or cyclic); (3) estrogen-only; (4) progestin-only; and (5) vaginal estrogen only. In secondary analyses, we examined associations between cumulative treatment duration (≤1 year; >1–4 years; >4 years) of continuous estrogen-progestin, cyclic estrogen-progestin, and progestin-only and risk of meningioma and glioma.

User status of hormone therapy was assessed in a separate analysis and defined according to the last treatment day prior to index date: (1) Current/recent 0 to 2 y (last treatment day within 2 years before index date); (2) previous >2–5 y (last treatment day within 2 to 5 years before index date); (3) previous >5–10 y (last treatment day within 5 to 10 years before index date); and (4) previous >10 y (last treatment day prior to 10 years before index date).

Systemic estrogen-only is used by women without a uterus. Thus, a separate analysis assessed the association between systemic estrogen-only use and meningioma or glioma risk in the clinical target population, i.e., hysterectomized women [22].

A lag time window before index date of 1 year (i.e., omitting any hormone therapy prescriptions within 1 year from diagnosis/matching) was consistently applied in all analyses to reduce the possibility of reverse causation bias [30]. All analyses were also performed with two-year lag time and without lag time as sensitivity analyses.

We conducted post hoc sensitivity analyses among women aged 50 to 55 years between 2000 and 2018. The oldest women in this subpopulation were 50 years old in 1995 (initiation of the prescription register), thus enabling a nearly complete assessment of exposure to menopausal hormone therapy around the age of menopause.

All analyses included educational level, anti-asthma drugs, and antihistamines as covariates and were conducted using R Statistical Software, R Core Team (2020) [31]. Two-sided *P*-values were calculated using Wald test. *P*-values <0.05 were considered significant.

This study is reported as per the Strengthening the Reporting of Observational Studies in Epidemiology (STROBE) guideline (S1 Checklist).

### Ethics statement

Danish law states that studies based on the national registers in Denmark do not require ethical approval or patient consent. This study was approved by the Danish Data Protection Agency (approval ID: P-2019-280) and the Danish Health Data Board (approval ID: FSEID-00005931).

## Results

**Fig 1:**
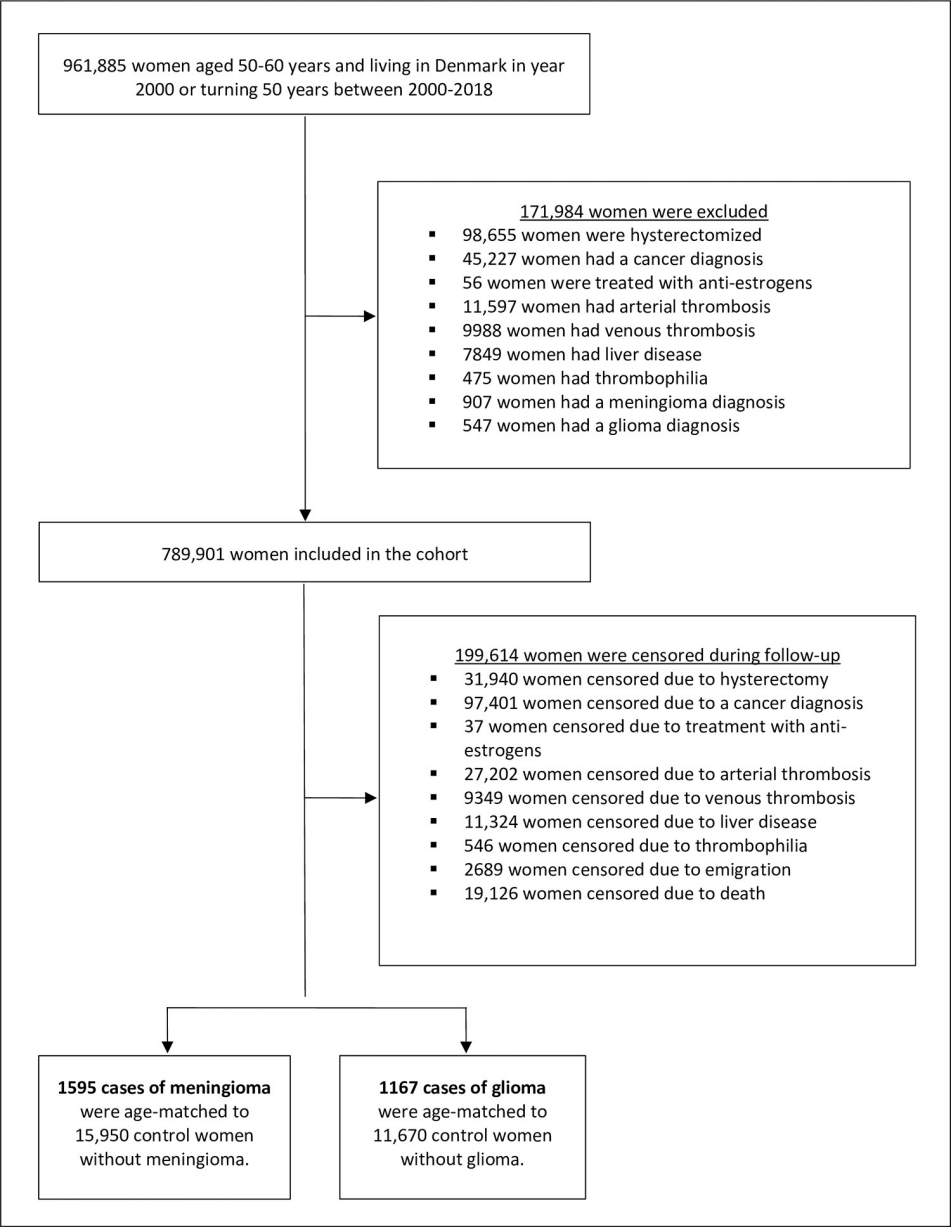
Flowchart of the establishment of the cohort and nested case-control populations.

The nationwide cohort included 789,901 eligible women followed for 8.5 million person-years with a median (first–third quartile) follow-up time of 10.8 years (5.0 years to 17.5 years). During follow-up, 1,595 (0.20%) women were diagnosed with meningioma and 1,167 (0.15%) with glioma. The establishment of the cohort and nested case-control populations are shown in Fig 1. Median age at time of diagnosis of both meningioma and glioma was 60 years (56 to 66 years). Characteristics of cases and controls in the 2 matched populations are specified in Table 1.

**Table 1:** Characteristics of the 2 matched population.

	Meningioma (*n* = 1,595)	Controls (*n* = 15,950)	Glioma (*n* = 1,167)	Controls (*n* = 11,670)	
**Age at diagnosis/matching—**median (years)	60 (56–66)		60 (56–66)		
**Follow-up time—**median (years)	8.9 (4.5–13.6)		8.1 (3.7–12.3)		
**Year of diagnosis/matching—**median	2013 (2008–2016)		2011 (2007–2015)		
**Hormone therapy use**					
Estrogen-progestin	423 (26.5)	3,785 (23.7)	277 (23.7)		2,827 (24.2)
Continuous	108 (25.5)	872 (23.0)	56 (20.2)		624 (22.1)
Cyclic	170 (40.2)	1,636 (43.2)	131 (47.3)		1,264 (44.7)
Mixed	129 (30.5)	1,098 (29.0)	76 (27.4)		771 (27.3)
Unknown	16 (3.8)	179 (4.7)	14 (5.1)		168 (5.9)
Estrogen only	33 (2.1)	284 (1.8)	21 (1.8)		223 (1.9)
Progestin only	143 (9.0)	1,203 (7.5)	99 (8.5)		849 (7.3)
Vaginal estrogen only	215 (13.5)	2,120 (13.3)	155 (13.3)		1,483 (12.7)
Estrogen only and progestin only	<4 (<0.3)	38 (0.2)	4 (0.3)		27 (0.2)
**Age at initiation—**median (years)					
Menopausal hormone therapy	50 (47–53)	50 (46–52)	50 (47–53)		50 (46–53)
Vaginal estrogen	56 (53–60)	56 (53–60)	56 (52–59)		56 (53–60)
**User status** (menopausal hormone therapy)					
Current/recent user 0–2 y	143 (23.8)	1,236 (23.3)	103 (25.7)		1,056 (26.9)
Previous user >2–5 y	85 (14.1)	752 (14.2)	56 (14.0)		602 (15.3)
Previous user >5–10 y	149 (24.8)	1,261 (23.7)	95 (23.7)		1,023 (26.1)
Previous user >10 y	230 (38.2)	2,150 (40.5)	152 (37.9)		1,312 (33.4)
**Educational level**					
Elementary school	473 (29.7)	5,225 (32.8)	407 (34.9)		3,875 (33.2)
Secondary school	45 (2.8)	424 (2.7)	32 (2.7)		299 (2.6)
Vocational education	668 (41.9)	6,459 (40.5)	476 (40.8)		4,621 (39.6)
University education	325 (20.4)	3,140 (19.7)	206 (17.7)		2,335 (20.0)
University and PhD	84 (5.3)	702 (4.4)	46 (3.9)		540 (4.6)
**Health-related variables**					
Diabetes	118 (7.4)	991 (6.2)	73 (6.3)		644 (5.5)
Asthma	530 (33.2)	5,011 (31.4)	366 (31.4)		3,474 (29.8)
Statins	397 (24.9)	3,259 (20.4)	238 (20.4)		2,199 (18.8)
Aspirin	247 (15.5)	1,893 (11.9)	154 (13.2)		1,267 (10.9)
Antihistamines	589 (36.9)	5,539 (34.7)	381 (32.6)		3,779 (32.4)
NSAIDs	1,268 (79.5)	12,217 (76.6)	914 (78.3)		8,794 (75.4)

Values either in number of women (%) or median (first–third quartile).

User status defined according to the last treatment day prior to index date: (1) Current/recent 0–2y—within 2 years before index date; (2) previous >2–5 y—within 2 to 5 years before index date; (3) previous >5–10 y—within 5 to 10 years before index date; and (4) previous >10 y—prior to 10 years before index date.

Users of combined estrogen-progestin comprised 26.5% of meningioma cases (23.7% of controls) and 23.7% of glioma cases (24.2% of controls), with a median age at treatment initiation of 50 years (47 to 53). Majority of menopausal hormone therapy users had their last treatment day earlier than 5 years before index date.

Among meningioma cases, 25.5% of users of estrogen-progestin were exposed to continuous progestin, while 40.2% were exposed to cyclic progestin. For glioma cases, the corresponding prevalences were 20.2% and 47.3%. Around a third of all estrogen-progestin users had either tried both continuous and cyclic treatment (27.3; 30.5%) or could not be reliably categorized (3.8; 5.9%).

Of all person-years of menopausal hormone therapy use in the cohort, 90.3% constituted oral administration, 7.9% transdermal administration, and 1.8% were used via other routes of administration (Table A in S1 Text). Estradiol was the primarily used type of estrogen (i.e., >96% of estrogen use). The most frequently used progestins in combined estrogen-progestin products were norethisterone (74%) and medroxyprogesterone (13%).

Progestin-only users comprised 9.0% of meningioma cases and 8.5% of glioma cases. Of the used progestin-only preparations, 68% contained medroxyprogesterone, 14% contained norethisterone, and 12% contained levonorgestrel (intrauterine device). Extensive details of hormone therapy usage in the cohort are specified in Table A in S1 Text.

**Fig 2:**
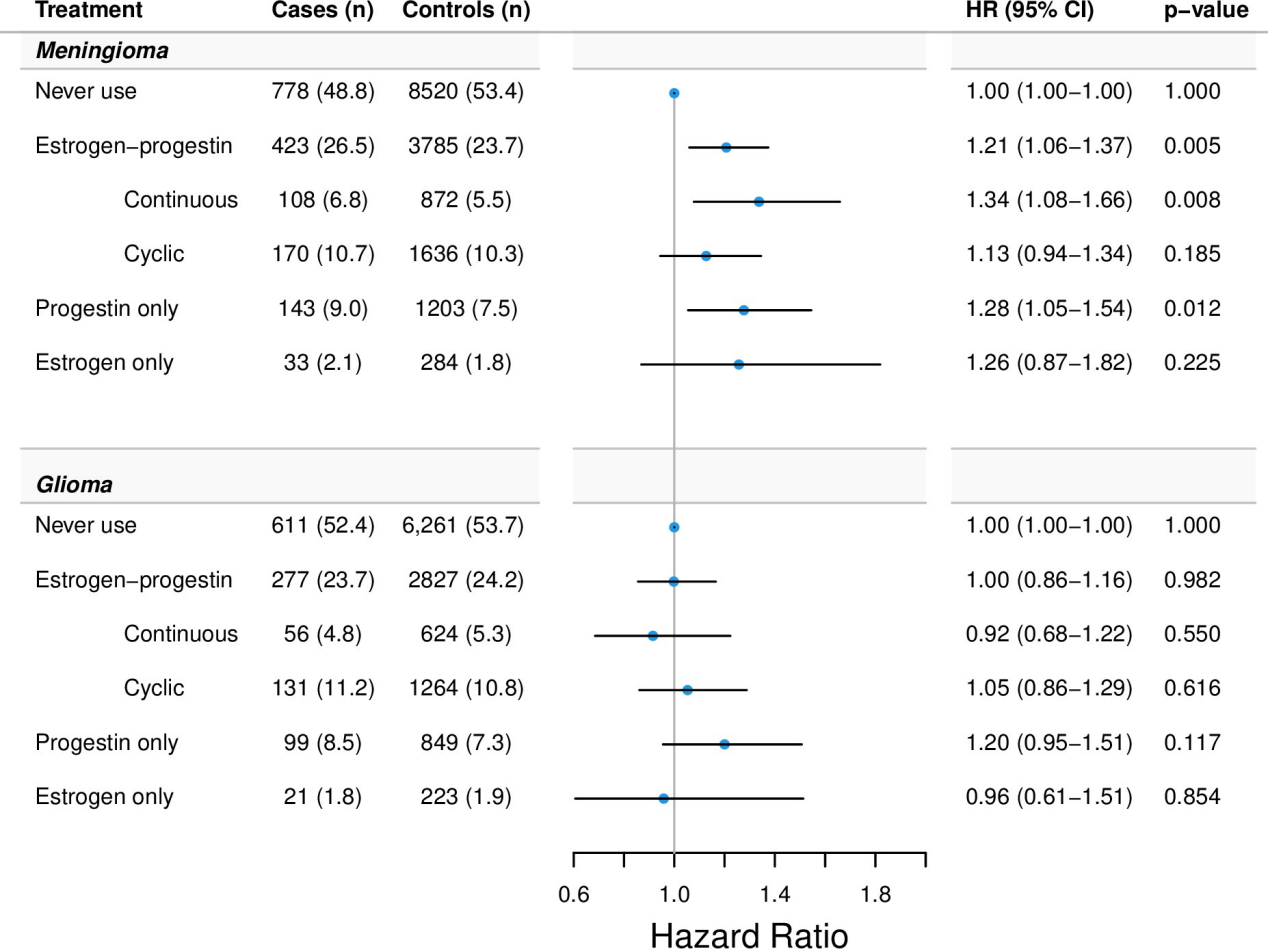
HRs of ever-use of hormone therapy types and association with CNS tumors. Footnote: In a separate cohort of hysterectomized women, exposure to estrogen-only yielded HR 1.22; (95% CI [0.90, 1.66] *p* = 0.20) for meningioma (122 cases and 1,179 controls) and 0.81; (95% CI [0.55, 1.18] *p* = 0.27) for glioma (65 cases and 632 controls) (Table B in S1 Text). Estimates for exposure to nonoverlapping estrogen-only and progestin-only, mixed or unknown estrogen-progestin therapy, and vaginal estrogen-only are shown in Table C in S1 Text. Adjusted for educational level and use of anti-asthma drugs and antihistamines. CI, confidence interval; CNS, central nervous system; HR, hazard ratio.

Ever-use of hormone therapy according to type and associations with meningioma and glioma are presented in Fig 2. Compared to never-use, use of estrogen-progestin was associated with an increased HR of meningioma 1.21; (95% CI [1.06, 1.37] *p* = 0.005), but not of glioma HR 1.00; (95% CI [0.86, 1.16] *p* = 0.982). Continuous estrogen-progestin was associated with a moderately increased HR for meningioma of 1.34; (95% CI [1.08, 1.66] *p* = 0.008). Cyclic treatment showed associations close to unity for both meningioma of 1.13; (95% CI [0.94, 1.34] *p* = 0.185) and glioma HR 1.05; (95% CI [0.86, 1.29] *p* = 0.616). Use of progestin-only was associated with slightly increased HRs for both meningioma 1.28; (95% CI [1.05, 1.54] *p* = 0.012) and glioma 1.20; (95% CI [0.95, 1.51] *p* = 0.117).

In the separate cohort of hysterectomized women, use of systemic estrogen-only yielded HR 1.22; (95% CI [0.90, 1.66] *p* = 0.203) for meningioma (122 cases and 1,179 controls) and HR 0.81; (95% CI [0.55, 1.18] *p* = 0.267) for glioma (65 cases and 632 controls) (Table B in S1 Text).

**Fig 3:**
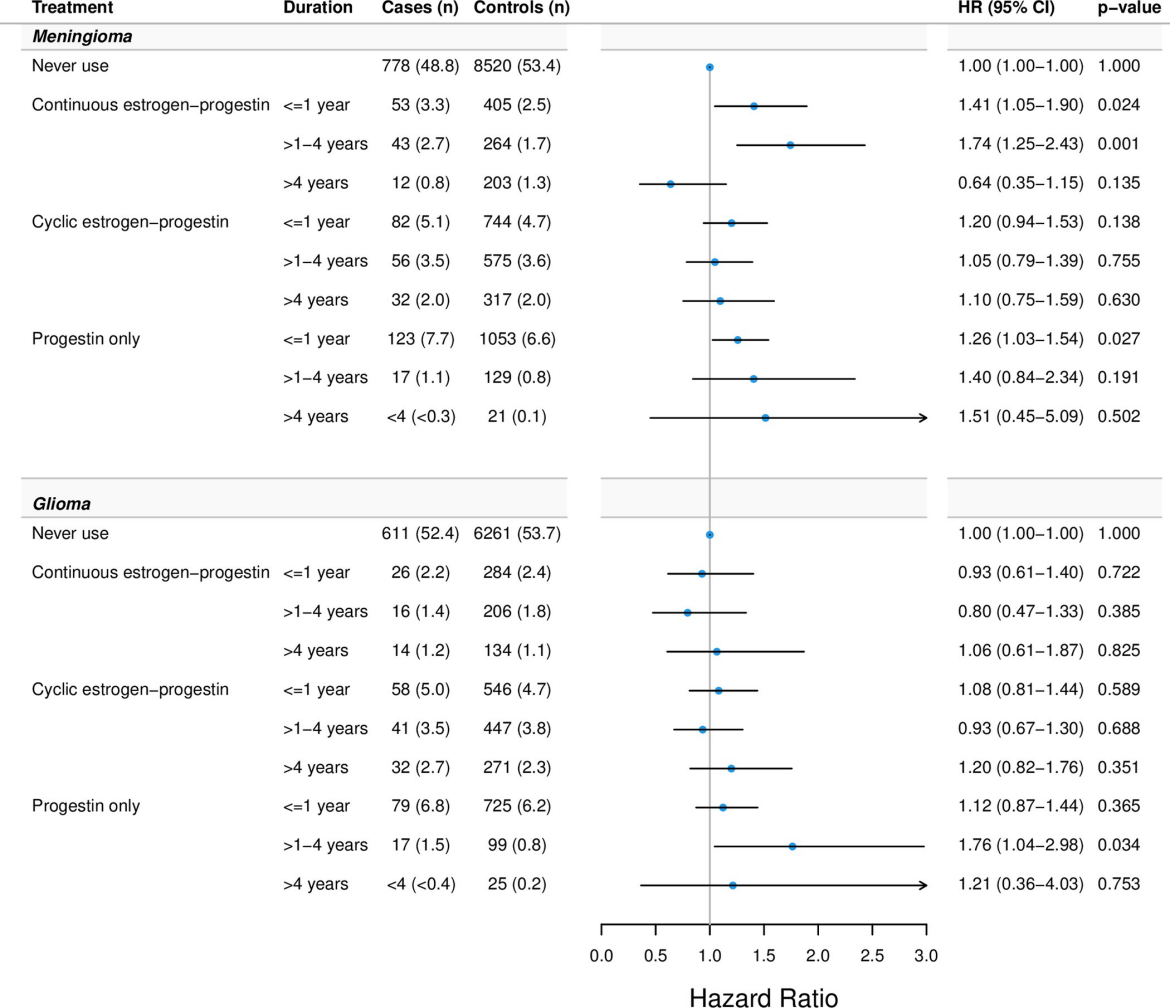
HRs of cumulative use of estrogen-progestin and progestin-only and association with CNS tumors. Footnote: Adjusted for educational level and use of anti-asthma drugs and antihistamines. CNS, central nervous system; HR, hazard ratio.

Fig 3 shows HRs for meningioma and glioma with cumulative estrogen-progestin use and progestin-only use. Cumulative use of continuous estrogen-progestin was associated with increased HRs for meningioma, but not in a consistent duration-response pattern. Cumulative use of cyclic estrogen-progestin was not associated with meningioma risk. Duration intervals of estrogen-progestin therapies were not associated with glioma. Although with limited statistical precision, use of progestin-only was associated with elevated risk estimates of meningioma that increased with longer cumulative duration of treatment. For glioma, no consistent duration-response pattern was seen with cumulative use of progestin-only.

**Fig 4:**
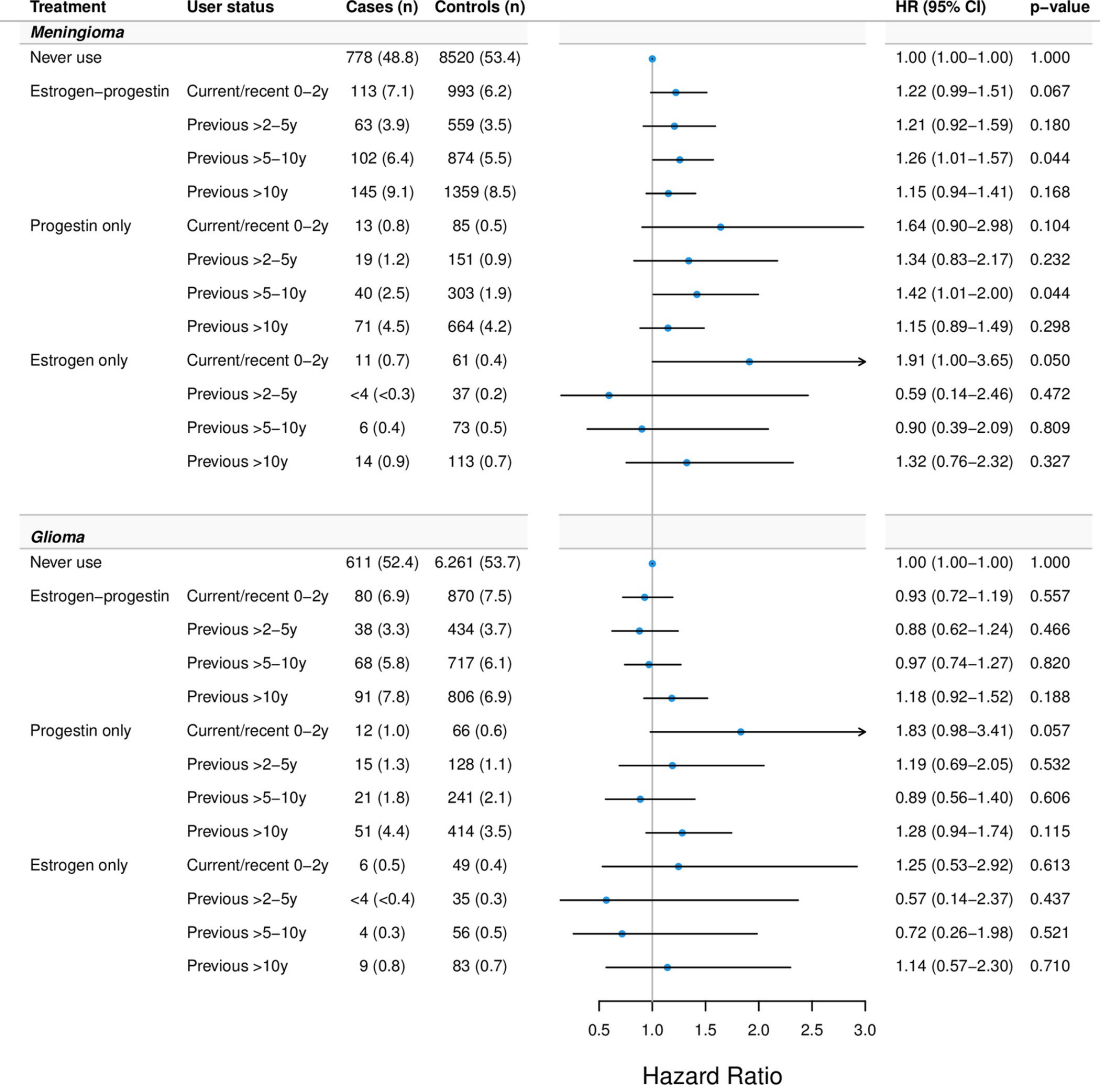
HRs of hormone therapy use and CNS tumors according to user status. Footnote: User status defined according to the last treatment day prior to index date: (1) Current/recent 0–2 y—within 2 years before index date; (2) previous >2–5 y—within 2 to 5 years before index date; (3) previous >5–10 y—within 5 to 10 years before index date; and (4) previous >10 y—prior to 10 years before index date. Adjusted for educational level and use of anti-asthma drugs and antihistamines. CNS, central nervous system; HR, hazard ratio.

Fig 4 shows associations for meningioma and glioma with estrogen-progestin use according to user status (current/recent versus previous), with previous use 5 to 10 years before index date showing the strongest association with meningioma HR 1.26; (95% CI [1.01, 1.57] *p* = 0.044). Current/recent use of progestin-only exhibited the highest HRs for both meningioma HR 1.64; (95% CI [0.90, 2.98] *p* = 0.104) and glioma HR, 1.83; (95% CI [0.98, 3.41] *p* = 0.057). User status of estrogen-only was not consistently associated with meningioma, but current/recent use yielded the highest estimate HR, 1.91; (95% CI [1.00, 3.65] *p* = 0.050).

Associations remained largely unchanged in sensitivity analyses with two-year lag time and without lag time (Figs A–F in S1 Text) as well as post hoc sensitivity analysis in a subpopulation with a nearly complete exposure history around the age of menopause (Table D in S1 Text).

## Discussion

In this nationwide population-based study nested in a cohort of peri- or postmenopausal women, use of estrogen-progestin was associated with increased risk of meningioma, while there was no evidence of increased glioma risk. The association for meningioma was restricted to continuous estrogen-progestin use. Previous use of estrogen-progestin, up to 10 years prior to the diagnosis, was associated with increased risk. Elevated risk estimates were also observed for use of progestin-only and risk of meningioma and possibly also glioma. Use of systemic estrogen-only was not associated with glioma risk, but a slightly increased risk for meningioma was seen for systemic estrogen-only therapy.

Receptors for both estrogen and progestin are present in meningiomas and gliomas, thus, exogenous exposure to female sex hormones could potentially have an impact on tumor growth [4,5]. The increased risk of meningioma observed among women using menopausal hormone therapy in previous studies has primarily been attributed to the estrogen component, as stronger associations have been found with use of estrogen-only [6,7] than with use of combined estrogen-progestin [6–9]. However, we found consistent associations for meningioma with continuous use of estrogen-progestin and additionally progestin-only use. Meningiomas express more progestin receptors than estrogen receptors (88% versus 40% in an immunohistochemical analysis [5]); hence, use of progestin can possibly influence the development of meningioma. Continuous combined hormone therapy includes a daily dosage of progestin, whereas cyclic therapies only include progestin in the end of a treatment cycle. We found that continuous estrogen-progestin therapy was consistently associated with an increased risk of meningioma, whereas cyclic estrogen-progestin therapy was not. These results are compatible with an observational study from 2013 based on Danish register data [8], that, although with limited statistical power, reported a slightly increased adjusted odds ratio (OR) (1.5; 1.0 to 2.2) for meningioma with ever-use of continuous estrogen-progestin but not with cyclic use (1.1; 0.7 to 1.6) [8]. Further, in line with the results of 2 recent observational studies (2020 and 2021) of exogenous progestin exposure and meningioma risk, we found an increased rate of meningioma among women using progestin-only preparations [14,15].

Continuous estrogen-progestin, as defined in this study, does not include the levonorgestrel releasing intrauterine device. Although, it can be used as the continuous progestin component in combined hormone therapy for postmenopausal women, we did not have sufficient statistical precision to examine the use of systemic estrogen combined with the levonorgestrel releasing intrauterine device and associations with meningioma. Thus, this should be assessed in future studies.

Our study results contribute to the understanding of the etiology of brain tumors with evidence highlighting the progestin component of hormonal therapy products as a potential risk factor for meningioma and possibly also glioma. Furthermore, although the absolute risks may be relatively small owing to the rarity of CNS tumors, our findings suggest cyclic estrogen-progestin as a safer therapy in relation to meningioma risk. The increasing body of evidence related to meningioma risk with female use of progestins calls for scientific attention [14,15,32].

A recent meta-analysis (2018) reported a decreased risk of glioma with menopausal hormone therapy use, but called for more studies with larger sample sizes to further explore the findings [10]. In line with a previous observational study, we found an overall neutral association between menopausal hormone therapy use and glioma risk [13].

Notably current/recent use of menopausal hormone therapy has been linked to an elevated risk of meningioma, whereas previous use has not exhibited an increased risk. However, most previous studies were based on relatively short follow-up periods [17,33]. In our study, we found an increased risk of meningioma among both current/recent users of estrogen-progestin and previous users (up to 10 years before the meningioma diagnosis). A possible explanation for the different findings regarding previous use is that extended follow-up time and larger sample sizes, such as in our study, is likely needed to examine the influence of exposure timing, crucial in evaluations of slowly progressing outcomes such as meningioma.

Many register-based studies investigating associations between use of menopausal hormone therapy and development of CNS tumors included women up to 75 to 89 years in their study populations [7,8,12,13,34]. Consequently, these studies failed to assess relevant exposure around the perimenopausal age for a significant proportion of included women, resulting in underestimation of the exposure in the study population and misclassification of users as non-users attenuating potential associations between use of menopausal hormone therapy and CNS tumor risk [7,8,12,13,34].

Strengths of the study included the large nationwide sample with long continuous follow-up, highly valid diagnoses of meningioma and glioma, and extensive details on hormone therapy use. The Danish prescription register provided complete data on filled prescriptions from 1995; therefore, only women aged 50 to 60 years between 2000 and 2018 were included. The age-restriction enabled detection of hormone therapy use around the menopausal age for most included women, thus, reducing bias towards the null present in many previous observational studies.

This study had limitations. First, only a small proportion of women in Denmark used transdermal hormone therapy and few were long-term users of progestin-only. Consequently, we could not evaluate the influence of modes of administration or long-term progestin-only use on the occurrence of CNS tumors. Associations between progestin use and CNS tumor risk are likely to differ with type of progestins. In our study, 74% of the total use of combined treatment contained norethisterone (13% contained medroxyprogesterone) and 68% of the total progestin-only use contained medroxyprogesterone (26% contained either norethisterone or levonorgestrel).

Second, since the prescription register initiated in 1995, we could not detect exposure to hormone therapy before 1995, possibly leading to an underestimation of the observed associations. Nonetheless, post hoc sensitivity analysis of women with a nearly complete history of exposure supported our main findings.

Similarly, information on past use of hormonal contraceptives before 1995 was not available. As such, potential confounding cannot be ruled out, given that former users of hormonal contraceptives were more likely to use menopausal hormone therapy.

Finally, owing to the observational nature of the study, we cannot exclude residual confounding from unmeasured or unknown risk factors for CNS tumors. However, many factors related to lifestyle have been thoroughly investigated and found not to impact the risk of meningioma or glioma [3]; thus, we do not expect residual confounding to have had a major influence on our findings. Further, we would expect the 2 outcomes in our study (i.e., meningioma and glioma) to largely share the same sources of residual bias and measured and unmeasured factors. Our finding that associations with meningioma and glioma were substantially different renders it possible that the difference was a result of the exposure (i.e., hormone therapy), thereby strengthening the likelihood of a causal link between menopausal hormone use and the outcomes.

By design, a nested case-control study with appropriate incidence density matching provides the same rate ratios as if the full cohort was analyzed prospectively [23,24]. However, absolute risk estimation was not possible in the nested case-control design.

In conclusion, use of estrogen-progestin was associated with an increased risk of meningioma, regardless of user status (current/recent versus previous), while there was no evidence of increased glioma risk. Continuous, but not cyclic, estrogen-progestin therapies were consistently associated with increased risk of meningioma. Progestin-only use was associated with risk of meningioma and potentially also glioma risk. Use of systemic or vaginal estrogen-only was not associated with glioma risk, but systemic estrogen-only may be associated with meningioma risk. Further studies are warranted to evaluate our findings and explore long-term progestin-only use and the risk of CNS tumors, since this study suggests a role of progestin in the etiology of CNS tumors.

## Supporting information

S1 Checklist STROBE statement. (DOC)

S1 Text Supporting information. (DOCX)

## Abbreviations

ATC: Anatomical Therapeutic Chemical
CI: confidence interval
CNS: central nervous system
HR: hazard ratio
OR: odds ratio
