# Supplementary material for: Menopausal hormone therapy and central nervous system tumors: Danish nested case-control study
Source: PLoS Med. 2023 Dec 19;20(12):e1004321. doi: 10.1371/journal.pmed.1004321 (PMC10729984; doi:10.1371/journal.pmed.1004321)
Supplement: S1 Text — (DOCX) [file pmed.1004321.s002.docx]

**Supporting Information**

**Table A**

All hormone therapy products (except vaginal estrogens) used by the study population including ATC-codes, active ingredients, mode of administration, unit size and the distribution of use among the 301,093 users in the cohort.

| **ATC-code** | **Active ingredient(s)** | **Mode of administration** | **Unit size (mg)** | **Unique users (%)** | **Total person-years used (%)** |
| --- | --- | --- | --- | --- | --- |
| **Estrogen-progestin products** | | | | | |
| **Monophasic drugs (continuous progestin)** | | | | | |
| G03FA01 | Estradiol + Norethisterone | Oral tablet | 1 Estrogen (E) + 0.5 Progestin (P)  2 E + 1 P | 9.66 | 12.78 |
| G03FA01 | Estradiol + Norethisterone | Transdermal patch | 0.05 E + 0.17 P (mg/24h) | 0.89 | 0.54 |
| G03FA11 | Ethinylestradiol + Levonorgestrel | Oral tablet | Unknown dose | 0.03 | 0.02 |
| G03FA12 | Estradiol + Medroxyprogesterone | Oral tablet | 1 E + 2.5 P  1 E + 5 P  2 E + 5 P | 1.09 | 0.81 |
| G03FA15 | Estradiol + Dienogest | Oral tablet | 2 E + 2 P | 0.42 | 0.27 |
| G03FA17 | Estradiol + Drospirenone | Oral tablet | 1 E + 2 P | 0.18 | 0.17 |
| **Multiphasic drugs (cyclic progestin)** | | | | | |
| G03FB01 | Estradiol + Norgestrel | Oral tablet | 28 days/2mg E + 10 days/0.5mg P | 0.90 | 0.99 |
| G03FB05 | Estradiol (+Estriol) + Norethisterone | Oral tablet | 28 days/1mg E + 12 days/1mg P  28 days/2mg E + 12 days/1mg P  (22 days/2mg + 6 days/1mg) E + 10 days/1mg P  *(22 days/4mg E2 + 2mg E3 + 6 days/1mg E2 + 0.5mg E3) + 10 days/1mg P  21 days/2mg E + 10 days/1mg P  (21 days/2mg + 7 days/1mg) E + 14 days/20mg P | 8.64 | 9.89 |
| G03FB05 | Estradiol + Norethisterone | Transdermal patch | 28 days/0.05mg/24h E + 14 days/0.17mg/24h P  28 days/0.05mg/24h E + 14 days/0.25mg/24h P | 1.59 | 1.07 |
| G03FB06 | Estradiol + Medroxyprogesterone | Oral tablet | 21 days/2mg E + 10 days/10mg P  22 days/2mg E + 10 days/5mg P  28 days/2mg E + 12 days/10mg P  84 days/2mg E + 14 days/20mg P | 3.49 | 3.36 |
| G03FB09 | Estradiol + Levonorgestrel | Oral tablet | 28 days/2mg E + 12 days/0.075mg P | 2.11 | 1.84 |
| G03FB11 | Estradiol + Trimegestone | Oral tablet | 28 days/2mg E + 14 days/0.5mg P | 0.12 | 0.05 |
| G03HB01 | Estradiol + Cyproterone | Oral tablet | 28 days/2mg E + 14 days/0.5mg P | 0.98 | 1.01 |
| **Estrogen-only products** | | | | | |
| G03CA03 | Estradiol | Nasal spray | 0.15 | 0.02 | <0.01 |
| G03CA03 | Estradiol | Transdermal patch | 0.025/0.0375/0.04/0.05/0.075/0.08/0.1 (mg/24h) | 3.80 | 4.37 |
| G03CA03 | Estradiol | Transdermal gel | 0.5/0.6/1/1.5 (mg/dose) | 2.05 | 1.96 |
| G03CA03 | Estradiol | Oral tablet | 1/2/4/5 | 18.97 | 43.70 |
| G03CA03 | Estradiol | Injection | 10 (mg/ml) | 0.04 | 0.06 |
| G03CA03 | Estradiol | Rectal suppository | 5 | <0.01 | <0.01 |
| G03CA04 | Estriol | Oral tablet | 1/2 | 0.54 | 0.43 |
| G03CA53 | Estradiol + Estriol | Oral tablet | *1 E2 + 0.5 E3  *2 E2 + 1 E3  *4 E2 + 2 E3 | 0.18 | 0.22 |
| G03CA57 | Conjugated estrogen | Oral tablet | 0.625/1.25 | 0.03 | 0.03 |
| G03CX01 | Tibolon | Oral tablet | 2.5 | 1.49 | 2.25 |
| **Progestin-only products** | | | | | |
| G03DA02 | Medroxyprogesterone | Injection | 25 (mg/ml) | 0.01 | <0.01 |
| G03DA02 | Medroxyprogesterone | Oral tablet | 5 | 14.07 | 9.69 |
| G03DA04 | Progesterone | Rectal suppository | 200 | 0.32 | 0.06 |
| G03DB01 | Dydrogesterone | Oral tablet | 10 | 0.01 | <0.01 |
| G03DB08 | Dienogest | Oral tablet | 2 | 0.42 | 0.36 |
| G03DC02 | Norethisterone | Oral tablet | 1/5 | 4.47 | 2.04 |
| G03DC03 | Lynestrenol | Oral tablet | 5 | 0.95 | 0.35 |
| G02BA03 | Levonorgestrel | Intrauterine device | 0.02 (mg/24h) | 2.27 | 1.64 |

**Multiple estrogen active ingredients; E2 = Estradiol, E3= Estriol*

**Table B**

HRs for CNS tumours with ever use of estrogen-only among hysterectomized women.

Analysis performed in two case-control populations nested in a separate cohort of hysterectomized women; i.e., 122 meningioma cases and 1179 controls; and 65 glioma cases and 632 controls.

| **Meningioma**  **(Hysterectomized women)** | | | | | |
| --- | --- | --- | --- | --- | --- |
| **Treatment** | **Cases** | **Controls** | **Hazard Ratio*** | **95% CI** | **P value** |
| Never use | 75 (26.5) | 873 (30.8) | 1.00 | (1.00-1.00) | 1.000 |
| Estrogen-only | 122 (43.1) | 1179 (41.7) | 1.22 | (0.90-1.66) | 0.203 |
|  |  |  |  |  |  |
| **Glioma**  **(Hysterectomized women)** | | | | | |
| **Treatment** | **Cases** | **Controls** | **Hazard Ratio*** | **95% CI** | **P value** |
| Never use | 58 (38.2) | 462 (30.4) | 1.00 | (1.00-1.00) | 1.000 |
| Estrogen-only | 65 (42.8) | 632 (41.6) | 0.81 | (0.55-1.18) | 0.267 |

**Adjusted for educational level, and use of anti-asthma drugs and antihistamines.*

**Table C**

HRs for CNS tumors with use of other hormonal therapy products not presented in the main figures

| **Meningioma** | | | | | |
| --- | --- | --- | --- | --- | --- |
| **Treatment** | **Cases** | **Controls** | **Hazard Ratio*** | **95% CI** | **P value** |
| Mixed estrogen-progestin | 129 (8.1) | 1098 (6.9) | 1.27 | (1.03-1.55) | 0.022 |
| Unknown estrogen-progestin | 16 (1.0) | 179 (1.1) | 0.97 | (0.58-1.62) | 0.900 |
| Estrogen-only & progestin-only | <4 (<0.3) | 38 (0.2) | 0.83 | (0.26-2.71) | 0.761 |
| Vaginal estrogen only | 215 (13.5) | 2120 (13.3) | 1.10 | (0.94-1.30) | 0.232 |
|  |  |  |  |  |  |
| **Glioma** | | | | | |
| **Treatment** | **Cases** | **Controls** | **Hazard Ratio*** | **95% CI** | **P value** |
| Mixed estrogen-progestin | 76 (6.5) | 771 (6.6) | 1.00 | (0.78-1.30) | 0.973 |
| Unknown estrogen-progestin | 14 (1.2) | 168 (1.4) | 0.86 | (0.50-1.49) | 0.593 |
| Estrogen-only & progestin-only | 4 (0.3) | 27 (0.2) | 1.51 | (0.52-4.34) | 0.446 |
| Vaginal estrogen only | 155 (13.3) | 1483 (12.7) | 1.08 | (0.89-1.30) | 0.459 |

**Adjusted for educational level, and use of anti-asthma drugs and antihistamines.*

**Table D**

HRs for CNS tumours with ever use of hormone therapy types among subpopulation of women aged 50-55 years between 2000-2018 (i.e., with a nearly complete exposure history)

| **Meningioma  (Women aged 50-55 years between 2000-2018)** | | | | | |
| --- | --- | --- | --- | --- | --- |
| **Treatment** | **Cases** | **Controls** | **Hazard Ratio*** | **95% CI** | **P value** |
| Never use | 611 (51.1) | 6,688 (55.8) | 1.00 | (1.00-1.00) | 1.000 |
| Estrogen-progestin | 281 (23.5) | 2575 (21.5) | 1.19 | (1.02-1.39) | 0.029 |
| Continuous | 66 (5.5) | 521 (4.3) | 1.36 | (1.04-1.79) | 0.027 |
| Cyclic | 124 (10.4) | 1225 (10.2) | 1.14 | (0.93-1.41) | 0.215 |
| Mixed | 79 (6.6) | 704 (5.9) | 1.19 | (0.92-1.54) | 0.191 |
| Unknown | 12 (1.0) | 125 (1.0) | 0.95 | (0.52-1.76) | 0.875 |
| Progestin only | 132 (11.0) | 1134 (9.5) | 1.25 | (1.02-1.53) | 0.029 |
| Estrogen only | 14 (1.2) | 169 (1.4) | 0.98 | (0.56-1.72) | 0.956 |
| Estrogen only & progestin only | <4 (<0.4) | 34 (0.3) | 0.91 | (0.28-2.99) | 0.880 |
| Vaginal estrogen only | 154 (12.9) | 1383 (11.5) | 1.22 | (1.01-1.48) | 0.042 |
|  |  |  |  |  |  |
| **Glioma  (Women aged 50-55 years between 2000-2018)** | | | | | |
| **Treatment** | **Cases** | **Controls** | **Hazard Ratio*** | **95% CI** | **P value** |
| Never use | 469 (53.7) | 4,791 (56.3) | 1.00 | (1.00-1.00) | 1.000 |
| Estrogen-progestin | 201 (23.0) | 1886 (22.2) | 1.06 | (0.88-1.27) | 0.558 |
| Continuous | 66 (5.5) | 521 (4.3) | 0.97 | (0.67-1.39) | 0.857 |
| Cyclic | 124 (10.4) | 1225 (10.2) | 1.10 | (0.87-1.38) | 0.445 |
| Mixed | 79 (6.6) | 704 (5.9) | 1.10 | (0.81-1.50) | 0.532 |
| Unknown | 12 (1.0) | 125 (1.0) | 0.84 | (0.44-1.63) | 0.615 |
| Progestin only | 92 (10.5) | 796 (9.4) | 1.18 | (0.93-1.50) | 0.173 |
| Estrogen only | 10 (1.1) | 119 (1.4) | 0.83 | (0.43-1.60) | 0.581 |
| Estrogen only & progestin only | 4 (0.5) | 24 (0.3) | 1.81 | (0.62-5.28) | 0.279 |
| Vaginal estrogen only | 97 (11.1) | 892 (10.5) | 1.08 | (0.85-1.38) | 0.535 |

**Adjusted for educational level, and use of anti-asthma drugs and antihistamines.*

**Fig A**

HRs of ever use of hormone therapy types and association with CNS tumors - Two-year lag-time window

*
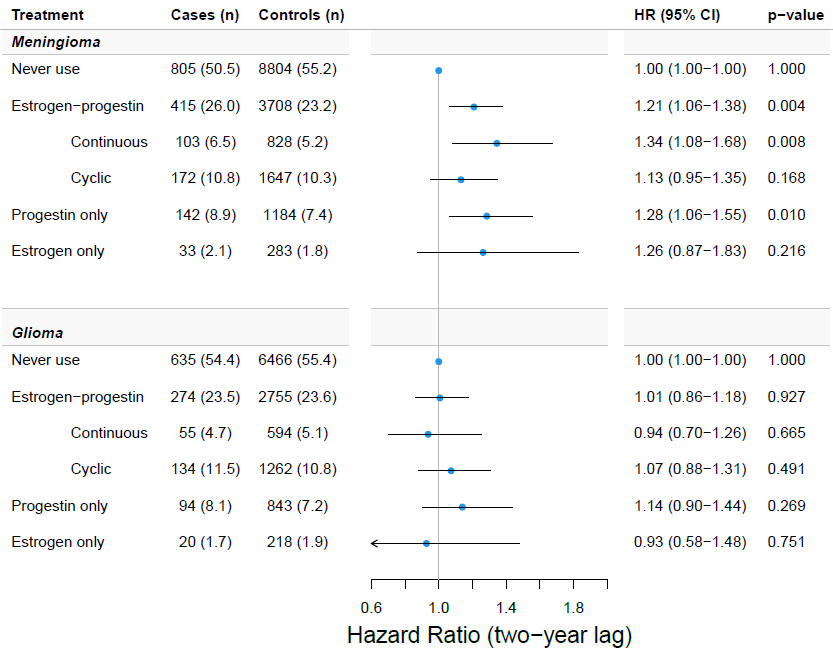

Adjusted for educational level, and use of anti-asthma drugs and antihistamines.*

**Fig B**

HRs of ever use of hormone therapy types and association with CNS tumors - No lag-time

*
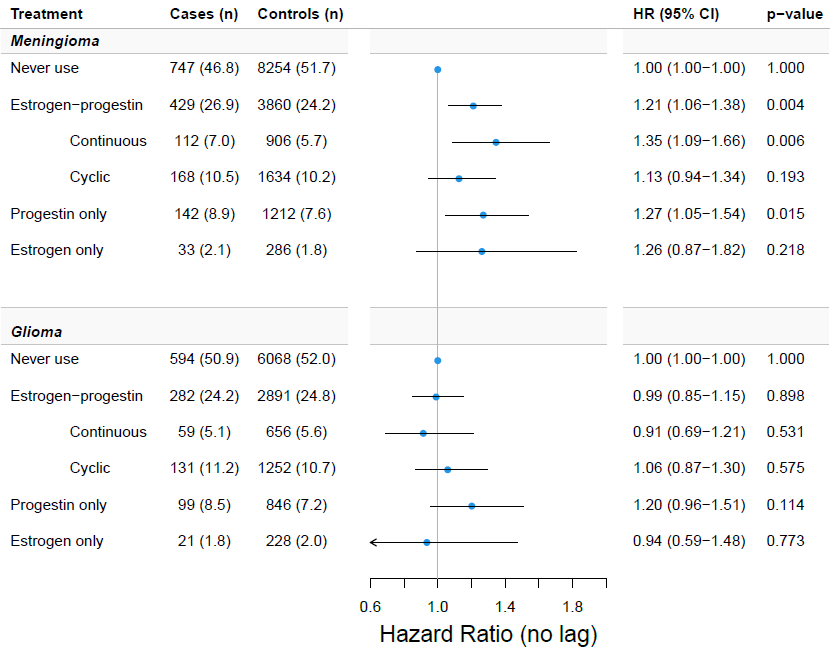

Adjusted for educational level, and use of anti-asthma drugs and antihistamines.*

**Fig C**

HRs of cumulative use of estrogen-progestin and progestin-only and association with CNS tumors - Two-year lag-time window


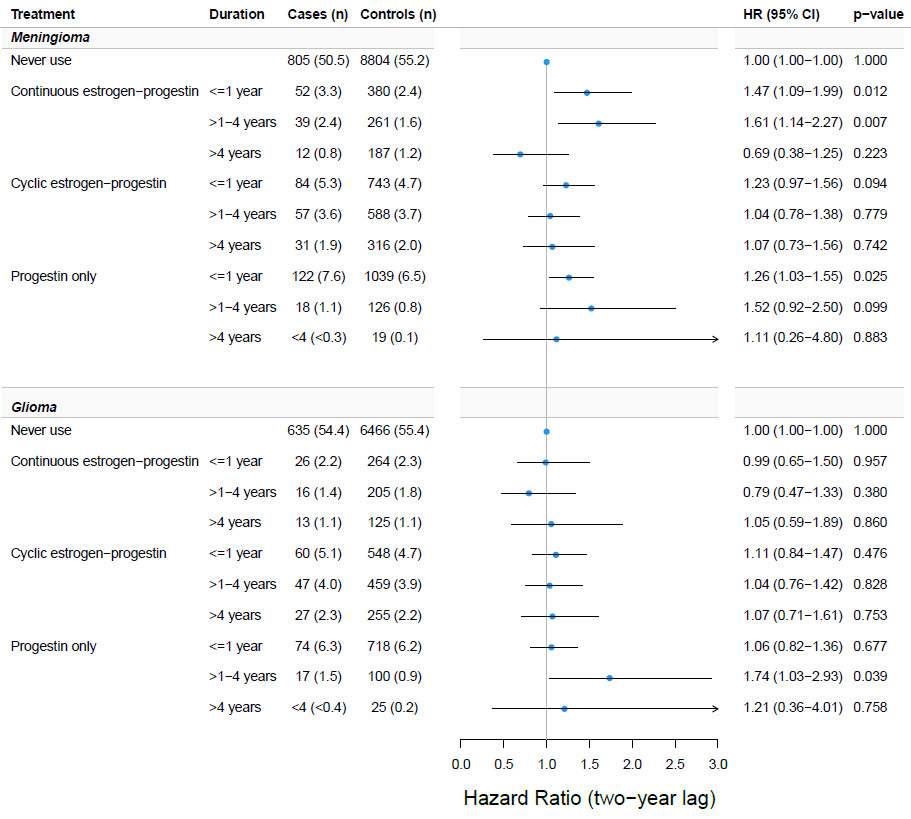


*Adjusted for educational level, and use of anti-asthma drugs and antihistamines.*

**Fig D**

HRs of cumulative use of estrogen-progestin and progestin-only and association with CNS tumors - No lag-time


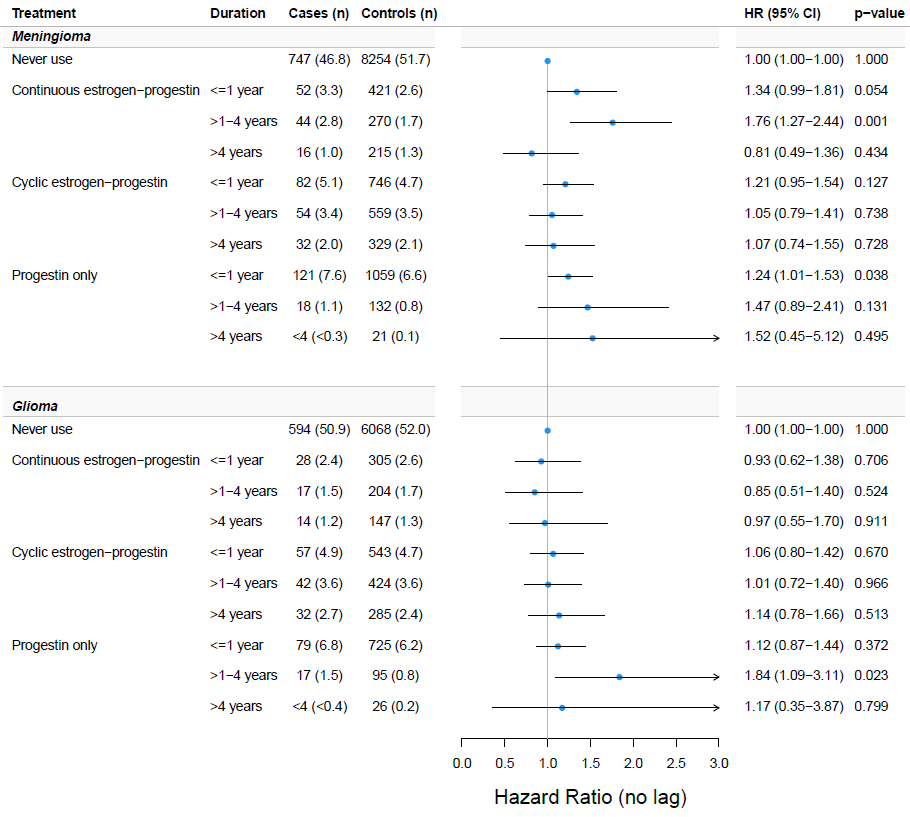


*Adjusted for educational level, and use of anti-asthma drugs and antihistamines.*

**Fig E**

HRs of hormone therapy use and CNS tumors according to user status - Two-year lag-time window

*
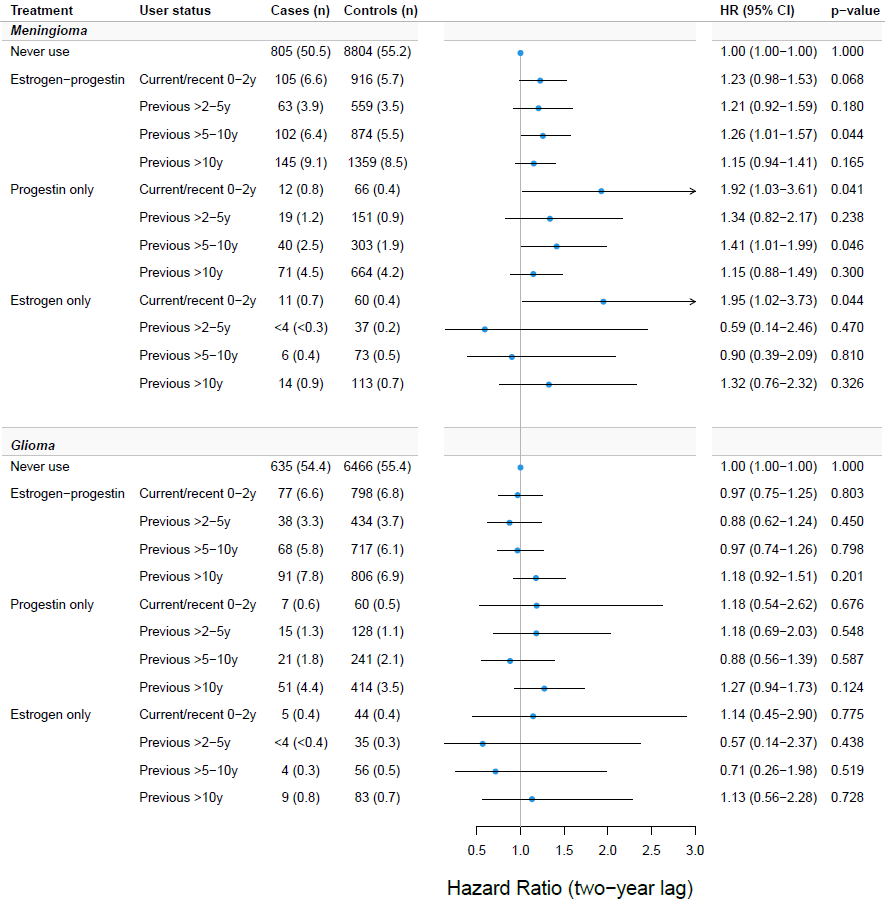
*

*User status defined according to the last treatment day prior to index date: 1) Current/recent 0-2y – within two years before index date; 2) Previous >2-5y – within two to five years before index date; 3) Previous >5-10y – within five to ten years before index date; 4) Previous >10y – prior to ten years before index date.*

*Adjusted for educational level, and use of anti-asthma drugs and antihistamines.*

**Fig F**

HRs of hormone therapy use and CNS tumors according to user status - No lag-time

*
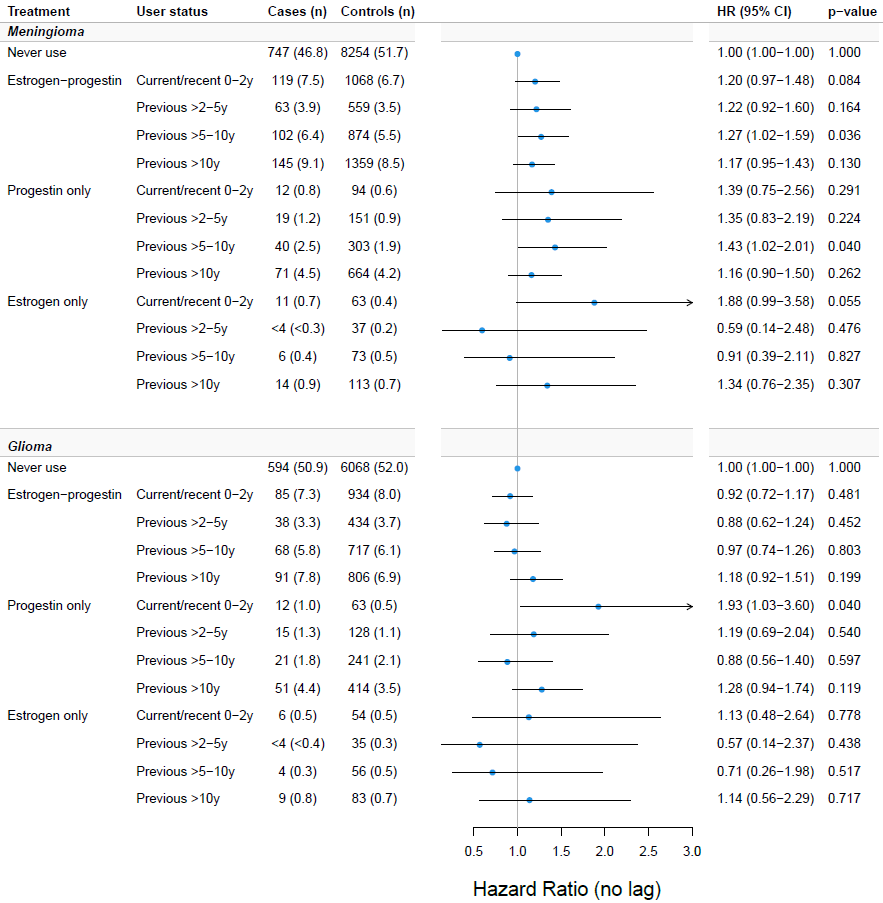
*

*User status defined according to the last treatment day prior to index date: 1) Current/recent 0-2y – within two years before index date; 2) Previous >2-5y – within two to five years before index date; 3) Previous >5-10y – within five to ten years before index date; 4) Previous >10y – prior to ten years before index date.*

*Adjusted for educational level, and use of anti-asthma drugs and antihistamines.*
